# Protocol for establishing spontaneous metastasis in mice using a subcutaneous tumor model

**DOI:** 10.1016/j.xpro.2024.103239

**Published:** 2024-08-02

**Authors:** Shiqin Liu, Michelle Shen, Kewei Le, Alifiani B. Hartono, Tanya Stoyanova

**Affiliations:** 1 Department of Molecular and Medical Pharmacology, University of California, Los Angeles, Los Angeles, CA 90095, USA; 2 Department of Urology, University of California, Los Angeles, Los Angeles, CA 90095, USA

**Keywords:** Cell Biology, Cancer, Model Organisms, Molecular Biology

## Abstract

Recapitulating spontaneous metastasis in preclinical models is crucial for understanding mechanisms underlying cancer progression and testing effective therapeutic interventions. We present a protocol for establishing and characterizing the spontaneous metastasis model in mice. We describe steps for generating primary tumors, tumor resection, monitoring metastatic dissemination, and evaluating metastatic burden using histological and imaging techniques. This protocol provides a valuable tool for studying metastasis *in vivo* and testing therapeutic strategies aimed at preventing or targeting metastatic diseases.

For complete details on the use and execution of this protocol, please refer to Liu et al.^1^

## Highlights

• Protocol to establish a clinically relevant spontaneous metastasis model
• Steps for generating primary tumor, tumor removal surgery, and evaluation of metastasis
• Instructions for organ collection and fluorescence imaging of the organs
• Guidance on histological and molecular biological analysis of organ tissue

## Abstract (editor highlights)

Publisher’s note: Undertaking any experimental protocol requires adherence to local institutional guidelines for laboratory safety and ethics.

## Graphical abstract

**Figure:**
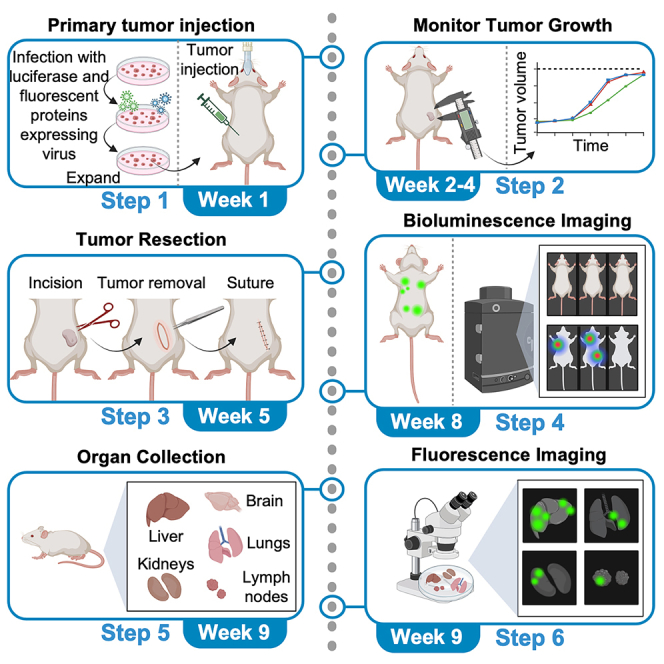

## Before you begin

### Metastasis model

The protocol describes the detailed steps to create a spontaneous metastasis model in immunocompromised mice for preclinical testing of gene function and therapeutic efficacy. Metastasis is associated with cancer mortality and represents a complex, multistep process involving tumor cell invasion, intravasation into the bloodstream or lymphatic system, circulation, extravasation, and colonization at distant sites.^2^^,^^3^^,^^4^ Despite advances in cancer research and treatment, metastatic disease remains a significant challenge due to its heterogeneity, spread to distant sites, and resistance to therapy.^5^^,^^6^ Therefore, there is a critical need for preclinical models that can recapitulate the process of metastasis to elucidate the mechanisms of cancer progression and test new effective therapeutic strategies.

Mouse models are widely used in cancer research due to their genetic and physiological similarities to humans as well as their amenability to genetic manipulation and experimental interventions. Various approaches have been developed to generate human metastasis in mice, including intracardiac injection metastasis model, orthotopic transplantation, intravenous injection model, intratibial injection model, and spontaneous metastasis models utilizing human cancer cells.^7^^,^^8^^,^^9^ The spontaneous metastasis model described in this protocol involves the implantation of tumor cells into the primary site, resulting in the formation of localized primary tumors and a natural progression to metastatic disease.

### Generation of luciferase-GFP/RFP-expressing UCHL1-positive cells

**Figure 1:**
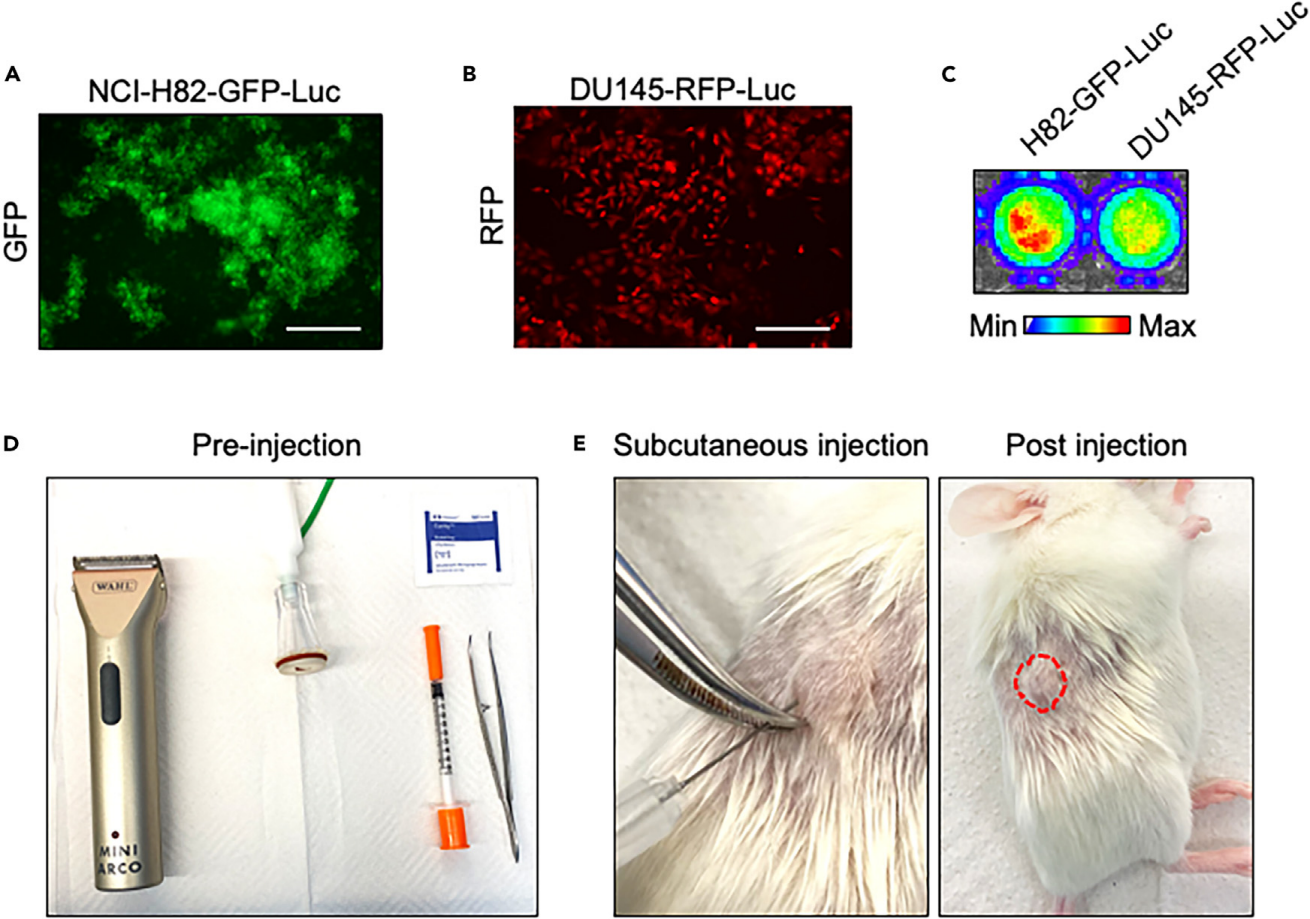
Establishing primary subcutaneous tumor (A and B) Fluorescence imaging of NCI-H82-GFP-luciferase cells and DU145-RFP-luciferase cells. Scale bars = 250 microns. (C) Bioluminescence imaging of the NCI-H82-GFP-luciferase cells and DU145-RFP-luciferase cells. (D) Example set-up of tumor cell implantation area. (E) Subcutaneous injection of NCI-H82-GFP-luciferase cells.

The protocol describes the specific steps for using two UCHL1-positive cell lines, NCI-H82 and DU145. We have also used this protocol in LNCaP and TD-NEPC^1^^,^^10^ cells. This protocol can also be applied to many other cancer cell lines. To track metastatic burden by bioluminescence imaging and fluorescence imaging, the cells indicated above are transduced with lentiviral vectors to stably express luciferase and green fluorescent protein (GFP) or red fluorescent protein (RFP) (Figures 1A–1C).

### Mouse strains

We use 6-8-week-old NSG (NOD-SCID-IL2Rγ–null) mice for *in vivo* models. We use male NSG mice for the prostate cancer model, and 1:1 ratio of female and male mice for the lung cancer model. Mice were housed for 2 weeks prior to experimentation at temperatures of 65–75°F with 40%–60% humidity at University of California, Los Angeles, animal facility. Veterinary care was provided by the Division of Laboratory Animal Medicine at University of California, Los Angeles.

### Institutional permissions

All animal experimental procedures performed in this study were approved by the University of California, Los Angeles, Administrative Panel on Laboratory Animal Care (APLAC), IACUC. All animal studies were conducted in accordance with the Animal Ethics Committee at UCLA.

### Cell preparation

**Timing: 1–2 h**

This step describes the preparation of cells expressing luciferase and fluorescent proteins for primary tumor subcutaneous injection.

1. Confirm the expression of luciferase and fluorescent proteins in cancer cells.
  a. Check the cells under the fluorescence microscope to confirm their expression of GFP or RFP (Figures 1A and 1B).
  b. Seed 50,000 cells/ well in a 96-well plate and culture the cells at 37°C (5% CO2) for 16 h.
  c. Aspirate cell culture media completely.
  d. Add 200 μl of media containing luciferin (150 μg/mL) in a 96-well plate.
  e. Incubate for 5 min at 37°C.
  f. Check bioluminescence by IVIS Lumina II (Figure 1C).
2. Cell counting.
  a. Culture cancer cell lines of the desired cancer type in an appropriate growth medium supplemented with 10% fetal bovine serum (FBS), 4 mM L-glutamine, and 1% penicillin-streptomycin. Examples are indicated in 2b and 2c below.
  b. Expand the DU145-RFP-luciferase in a 150 mm petri dish.
  c. Expand the NCI-H82 GFP-luciferase in a T25-flask.
    ***Note:*** Suspension cells are cultured in T25-Flasks. Ensure culture of sufficient number of cells based on the experimental design and number of animals to be injected.
  d. Cell dissociation for attached cells.
    i. Aspirate the cell culture medium.
    ii. Wash the cells with 2 mL 1x PBS once.
    iii. Aspirate the PBS.
    iv. Add 5 mL 0.05% Trypsin and incubate the cells for 5 min in 5% CO_2_ at 37°C.
    v. Collect the cells in the falcon tube with 5 mL culture medium.
  e. Cell dissociation for suspension cells.
    i. Collect the cell culture media and cells in a 15 mL Falcon tube.
    ii. Pellet the cells by centrifuge at 500 *g* × 3 min at 20–22°C.
    iii. Aspirate the medium.
    iv. Wash the cells with 2 mL 1x PBS once.
    v. Pellet the cells by centrifuge at 500 *g* × 3 min at 20–22°C.
    vi. Aspirate the PBS.
    vii. Add 5 mL 0.05% Trypsin and incubate the cells for 5 min in 5% CO_2_ at 37°C.
    viii. Collect the cells in the 15 mL Falcon tube with 5 mL culture medium.
  f. Centrifuge the cells at 500 *g* × 3 min at 20–22°C.
  g. Resuspend the cells in 5 mL medium.
  h. Take 10 μl of the cells to mix with 10 μl of Trypan blue.
  i. Count the cells.
    **CRITICAL:** Accurate cell count is critical. Only counts the live cells. The live cell percentage needs to be more than 90% before injection. Approximately 20 × 10^6^ cells are expected at 90% of confluency in a 150 mm tissue culture dish. Approximately 3 × 10^6^ cells are expected at 90% of confluency in a T-25 flask.
3. Resuspend 1 × 10^6^ cells in 80% Matrigel with culture medium to make the final volume 100 μL for each tumor implantation.
4. Store the tube on ice, less than 30 min.

**CRITICAL:** Aliquot 1 × 10^6^ cells mixed with 80% Matrigel in an individual Eppendorf tube for a single tumor implantation. Always keep the Matrigel on ice. Implant the cells immediately after harvesting the cells. Alternatively, Geltrex can be used for primary tumor implantation.

### Animal work items preparation

**Timing: 30 min**

This section describes the preparation of animal tumor injection tools and surgical area.

5. Preparation for subcutaneous tumor injection (Figure 1D).
  a. Autoclave forceps.
  b. Prepare insulin syringes (29G) for subcutaneous tumor injection.
  c. Ethanol pad.
  d. Pipette and pipette tips.
  e. Anesthesia system.
  f. Shaver.
6. Autoclave surgical tools including.
  a. Forceps.
  b. Scissors.
  c. Needle holder.
7. Prepare the tools for surgery including.
  a. Cauterizer.
  b. Absorbable sutures.
  c. Heat pads.
  d. Ophthalmic ointment.
  e. Diet-gel.
  f. Medi-gel.
  g. Carprofen.
8. Prepare the animal work area.
  a. Spray and wipe the surgical area with 70% ethanol.
  b. Put a clean surgical pad.
  c. Set up anesthesia system including chamber and nosecone.
  d. Spray and wipe the anesthesia chamber and nosecone with 70% ethanol.
9. Set up all surgical tools and items in the surgical area.

***Note:*** All animal procedure needs sterile conditions.

## Key resources table

**Table:** 

REAGENT or RESOURCE	SOURCE	IDENTIFIER
**Antibodies**		

Mouse anti-human-Ki67 (1:100)	Santa Cruz Biotechnology	Cat#sc-23900
Mouse anti-human-UCHL1 (1:100)	Santa Cruz Biotechnology	Cat#sc-271639
Mouse anti-human-SYP (1:100)	Santa Cruz Biotechnology	Cat#sc-17750
Mouse anti-human-NCAM (1:100)	Santa Cruz Biotechnology	Cat#sc-7326
Mouse anti-dsRed (1:100)	Santa Cruz Biotechnology	Cat#sc-390909

**Bacterial and virus strains**		

FUCGW	University of California, Los Angeles	Owen Witte
FUCRW	University of California, Los Angeles	Owen Witte
pHIV-LucZsGreen	Bryan Welm et al.	Addgene plasmid # 39196; http://n2t.net/addgene:39196; RRID: Addgene_39196

**Chemicals, peptides, and recombinant proteins**		

Formalin	Fisher Scientific	Cat#23-730-581
D-luciferin	PerkinElmer	N/A
Carprofen	Zoetis	N/A
Matrigel	Corning	Cat#CB-40234
Geltrax	Gibco	Cat#A1413202
DAB	Dako	Cat#K346811-2
Hematoxylin	Sigma-Aldrich	Cat#GHS316
Eosin	Fisher Scientific	Cat#E511-25

**Critical commercial assays**		

IHC kit	Promega	Cat#G8081

**Experimental models: Cell lines**		

DU145	ATCC	Cat#HTB-81
NCI-H82	ATCC	Cat#HTB-175

**Experimental models: Organisms/strains**		

NOD-SCID-IL2Rγ-null (male and female, 6–8 weeks)	Jackson Laboratory	NOD-SCID-IL2Rγ–null

**Software and algorithms**		

ImageJ	ImageJ	https://imagej.net/Downloads
Prism (version 10)	GraphPad	https://www.graphpad.com/scientific-software/prism/

**Other**		

BioRender		https://biorender.com/
DMEM media	Gibco	Cat#12-800-017
RPMI media	Gibco	Cat#31-800-022
Trypsin/EDTA (0.25%)	Gibco	Cat#15-400-054
Penicillin/Streptomycin	Gibco	Cat#15-140-122
GlutaMAX	Gibco	Cat#35-050-061
Cell counting slides	Invitrogen	Cat#C10228
Countess 3 automated cell counter	Thermo Fisher Scientific	N/A
Gemini cautery system	Harvard Apparatus	Cat#53153
6-0 coated vicryl sutures (absorbable)	Fisher Scientific	Cat#50-118-0849
29G insulin syringe	Fisher Scientific	Cat#14-841-33
Eye ointment	Bausch + Lomb	Cat#24208-780-55
Forceps	Fine Science Tools	Cat#11051-10
Scissors	Fine Science Tools	Cat#14060-09
Halsey needle holder	Fine Science Tools	Cat#10003-12
MediGel	Clear H_2_O	Cat#74-02-5022
DietGel	Clear H_2_O	Cat#72-06-5022
Stereo microscopes	Leica	Cat#M205
IVIS Lumina II	PerkinElmer	N/A

## Step-by-step method details

### Generate primary tumors

**Timing: 10 min per mouse**

This section describes the steps of implanting subcutaneous primary tumors using the NCI-H82-GFP-Luciferase positive cell line.

1. Anesthetize mice using isoflurane or a suitable alternative.

***Note:*** Mice are under anesthesia with 2% isoflurane delivered with 8% oxygen. Anesthetic monitoring can be accessed by toe pinch, foot pad color monitoring, and breathing.

2. Remove hair from the right dorsal flank of the mouse.
3. Position the mouse on its left side with the right dorsal flank facing upward.

***Note:*** Implant a single tumor on one side of the dorsal flank of the mouse. Keep all the primary tumors on the same side. Choose the 4^th^ pair of mammary fat pads for breast cancer models and the appropriate tissue sites for other cancer types.

4. Disinfect the skin with 70% ethanol pad.
5. Resuspend the cells with a pipette.

**CRITICAL:** Ensure to mix the cells very well before taking the cells with a syringe. Avoid generating bubbles while mixing. Do not vortex the cells.

6. Collect the cells (100 μL) in a pre-chilled 29G insulin syringe.

**CRITICAL:** We use 100 μL for mice with average weight from 25‒30 g. The volume of the cells can be adjusted based on animal size between 50‒100 μl. Too less volume may cause more variation of the size of the subcutaneous tumor. Put the syringe on ice for 5 min to pre-chill to avoid solidification of Matrigel.

7. Lift the skin with sterile forceps and insert the insulin syringe subcutaneously (See an example in Figure 1E).
8. Pull back gently on the plunger of the syringe to check that there is no blood.
9. Inject tumor cells slowly.
10. Wait for 2 min.

**CRITICAL:** It is imperative that the needle is not inserted too deeply. Ensure the injection of the tumor cells is subcutaneous. Give 2 min for the Matrigel to solidify.

11. Monitor mice for recovery and provide postoperative care as needed.

### Monitor primary tumor growth

**Timing: 2–4 weeks**

This section describes the assessment of subcutaneous primary tumor growth by caliper measurement.

**Figure 2:**
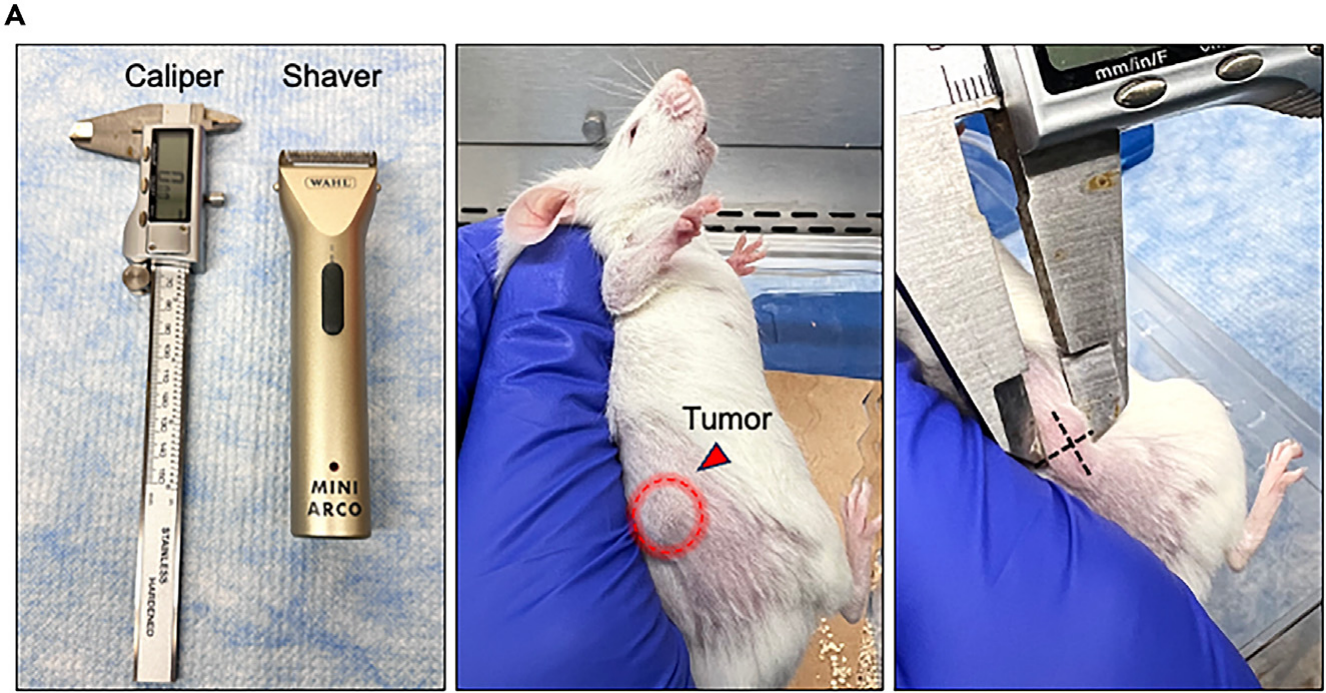
Evaluation and monitoring of primary tumor burden (A) Example set-up of tumor measurement and measurement of tumor volume by caliper.

***Note:*** Prepare the caliper and shaver for measurement (Figure 2).

12. Monitor primary tumor growth regularly by palpation.
13. Start measuring the primary tumor when it is palpable.

***Note:*** Tumor is usually palpable 2–3 weeks after implantation of 1 × 10^6^ cancer cells. The timeline of measuring the primary tumor varies based on different cancer types and different cell lines (troubleshooting 1).

14. Gently shave the hair surrounding the tumor site.
15. Restrain the mouse.
16. Measure the height, width, and length of the tumor by caliper (Figure 2).
  a. The equation of the tumor size can be calculated: tumor volume = (height × width × length) / 2.
  b. If the tumor is implanted in the mammary fat pat, or somewhere only the width and length can be measured, calculate tumor volume using the formula: tumor volume = (length × widthˆ2) / 2.

### Tumor removal surgery

**Timing: 15 min per mouse**

This part of the protocol describes the surgery to remove the primary tumor. The tumor resection is scheduled when the average primary tumor volume reaches 400 mm^3^.

***Note:*** Limited spontaneous metastasis or no metastasis can be detected if the primary tumor size is too small (<200 mm^3^). It is more challenging to remove the tumor if the primary tumor size is over 1.0 cm^3^.

17. Surgical preparation of the animal (Methods video S1).
  a. Anesthetize mice using isoflurane or a suitable alternative.
  b. Place ophthalmic ointment on both eyes.
  c. Inject carprofen (5 mg/kg) via intraperitoneal injection with an insulin syringe.
  d. Position the mouse on its left side with the tumor facing upward.
  e. Keep the mouse on top of the heat pad during the procedure.
  f. Check their response to stimuli to assess the depth of anesthesia.
  g. Remove hair surrounding the tumor site.
  h. Sterilize the surgical area with Iodine followed by 70% Ethanol swabs.
  i. Repeat the step of sterilization three times.
  j. Cover the animal except the surgery site with a sterile drape.

**CRITICAL:** All procedures should be performed according to the Administrative Panel on Laboratory Animal Care Guidelines for Rodent Survival Surgery.

**Figure 3:**
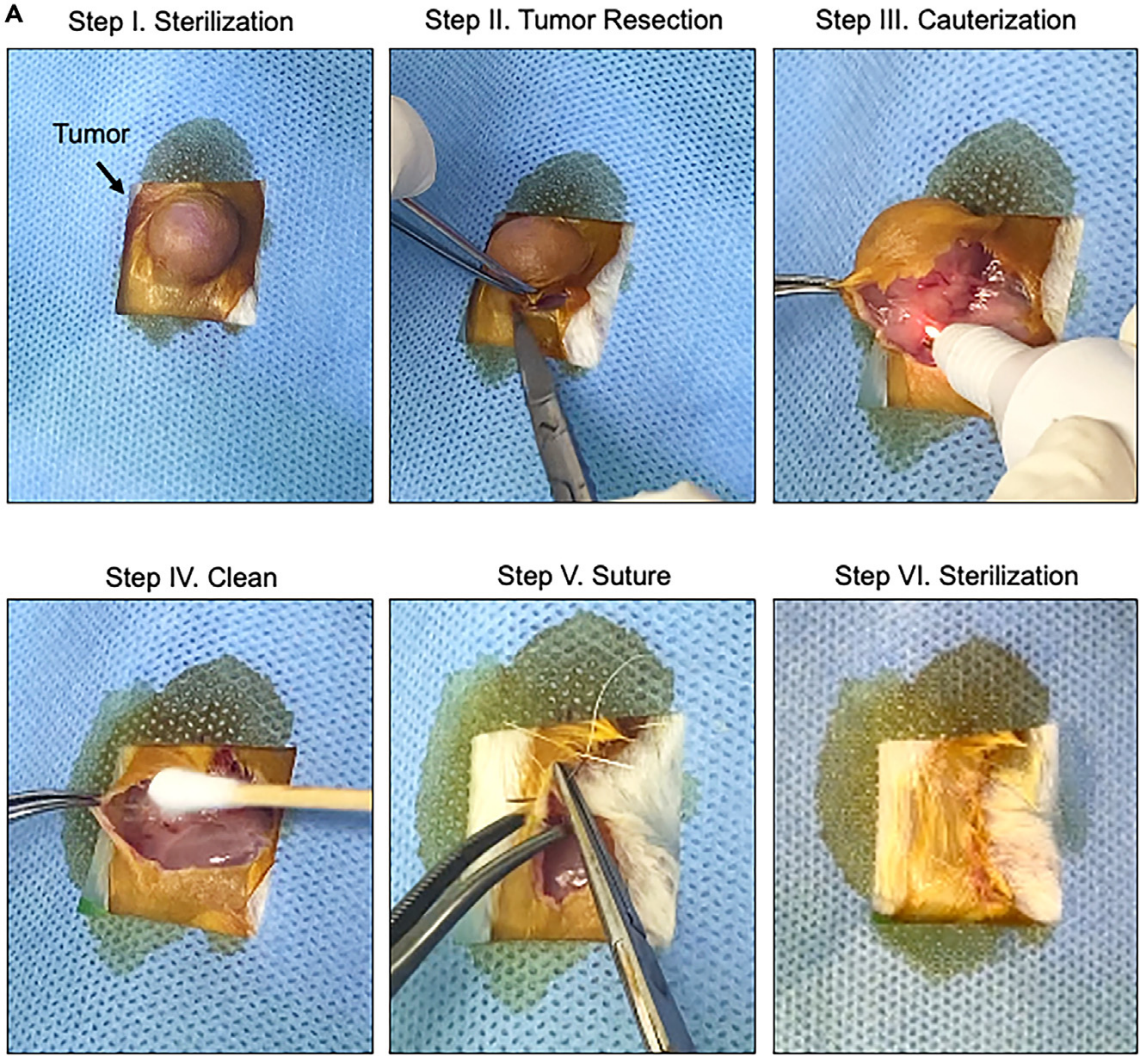
Tumor removal surgery (A) detailed steps of tumor removal surgery including preparing the surgical area, tumor resection, cauterization of blood vessels, stop bleeding, suture, and last sterilization.

18. Tumor removal surgery (Figure 3, and Methods video S1).
  a. Use sterile technique to make a 5–8 mm incision through the skin on the right side of the tumor.
  b. Use scissors to cut the skin surrounding the tumor by lifting the skin with blunt forceps.
  c. Using sterile scissors and forceps, gently separate the tumor from the subcutaneous layers.
    **CRITICAL:** Avoid cutting too deep to prevent damaging major organs.
  d. Cauterize any capillaries or blood vessels stemming from the tumor to prevent bleeding.
  e. Completely remove the primary tumor and the skin attached to the tumor.
  f. Check the surgical area to ensure complete removal of the primary tumor.
  g. Ensure no bleeding in the surgical area.
  h. Suture and close the surgical area with sterile 6–0 absorbable sutures.
    ***Note:*** Alternatively, the surgical area can be closed with sterile 7 mm wound clips.
  i. Sterilize the surgical area.
  j. Put the mouse in the recovery area.
  k. Monitor the mouse condition after surgery and provide postoperative care as needed (troubleshooting 2).
  l. At 24 h post-surgery, provide the mouse with a Medi-gel cup.
  m. At 48 h post-surgery, feed the mouse with a diet-gel cup.
  n. Actively monitor the mouse post-surgery.
    ***Note:*** Wound clips need to be removed at 7 days post-operatively.
  o. Process the primary tumor.
    i. Fix the primary tumor immediately after surgery in 10% neutral buffered formalin for 24 h at 4°C for 16 h.
    ii. Replace the formalin with 70% Ethanol.
    iii. Process the tumor and embed it with paraffin for histopathological analysis.

Methods video S1. Tumor removal surgery, related to step 18

### Monitoring metastatic dissemination

**Timing: 10 min per mouse**

This step describes monitoring spontaneous metastasis by mice behavior and whole-body bioluminescence imaging.

**Figure 4:**
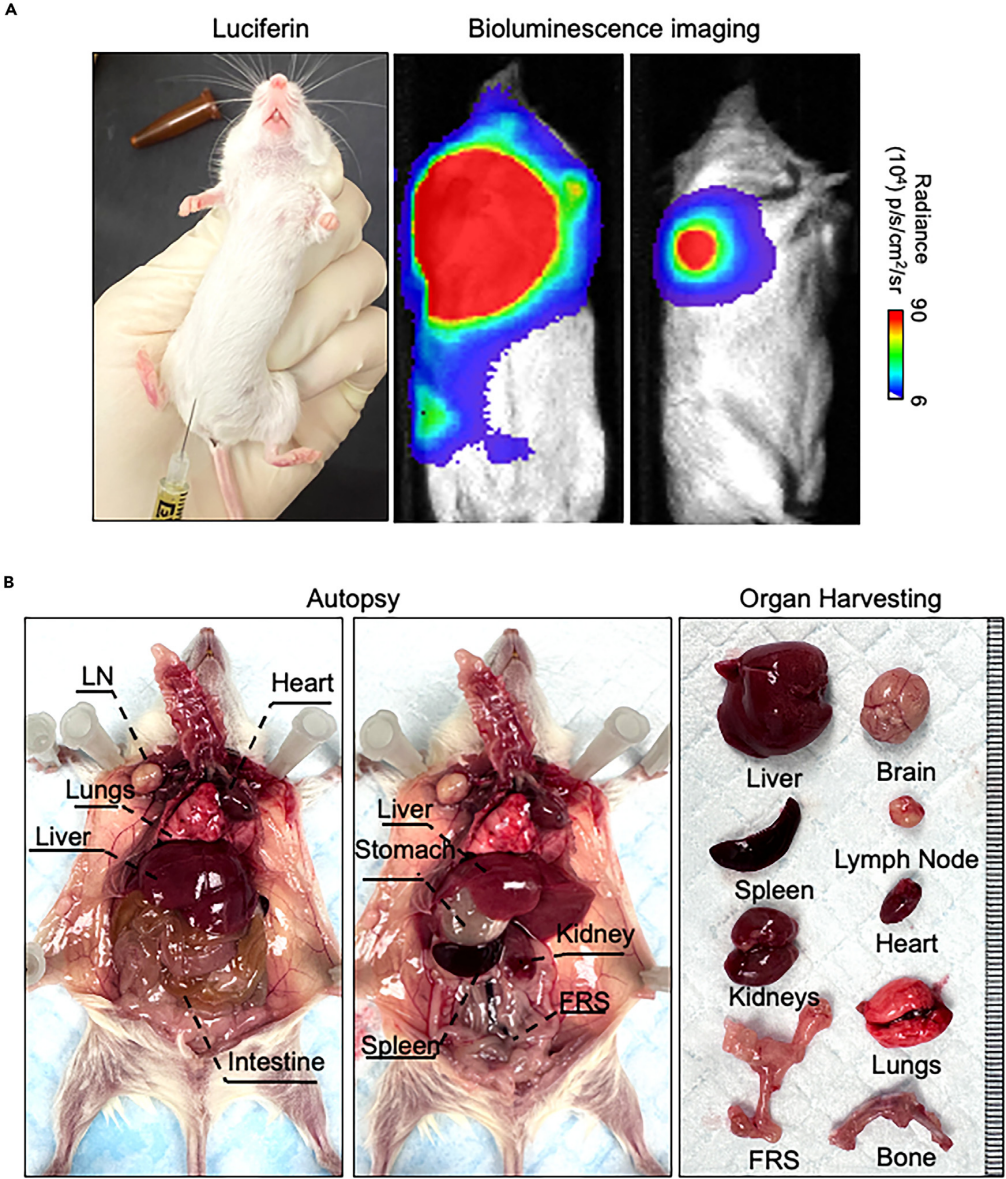
Whole body bioluminescence imaging and metastatic organ harvesting (A) Intraperitoneal (IP) injection of luciferin and bioluminescence imaging of mice with spontaneous metastasis. (B) An example of harvesting organs from mice with spontaneous metastasis.
  c. Imaging the mice by IVIS Lumina II (Figure 4A).
    i. Initiate the machine.
    ii. Place the mouse in the machine with proper anesthesia.
    iii. Select the luminescence channel.
    iv. Image mice from 1 min to 4 min to detect luminescence signals.
      ***Note:*** Different mouse imaging positions can help improve the detection of luminescent signals according to major metastatic sites. The main metastatic organs vary depending on different cancer type models. The major metastatic sites of prostate cancer model (DU145) are the lungs, lymph nodes, and kidneys. The metastatic organs of small cell lung cancer model (NCI-H82) include lymph nodes and liver.
  d. Recover the mice from anesthesia.
  e. Perform bioluminescence imaging once a week.
  f. Quantify and record the bioluminescence signals every week.
    ***Note:*** The experimental endpoint varies based on bioluminescent signals, metastatic burden, and mouse health status. In this protocol, the endpoint of the prostate cancer model and small cell lung cancer model is 3 weeks after surgical removal of the primary tumor. Mice should be imaged once a week to actively monitor the metastasis burden according to the bioluminescence signals. Based on our previous experience, the good timing of ending the experiments is when the average bioluminescence signals reach between 10^5^ and 10^6^ Photo photons/second/cm2/surface radiance.

19. Monitor mice for signs of metastatic dissemination (troubleshooting 3). This includes.
  a. Weight loss.
  b. Reduced activity.
  c. Reduced (or labored) food and drink consumption.
  d. Lethargy.
  e. Palpable masses at distant sites.
20. Conduct regular bioluminescence imaging once a week post-surgery to detect metastatic lesions (troubleshooting 4).
  a. Anesthetize mice using isoflurane or a suitable alternative.
  b. Inject luciferin (150 mg/kg) via intraperitoneal injection (Figure 4A).

### Evaluate spontaneous metastasis

**Timing: 1 week**

This section describes the assessment of spontaneous metastasis by harvesting metastatic organs for fluorescence imaging and histopathological analysis. This can provide a more quantifiable and precise evaluation of spontaneous metastasis by number, size, and staining intensity.

21. Organ collection (Figure 4B).
  a. Euthanize mice by CO_2_ inhalation followed by cervical dislocation at the experimental endpoint.
  b. Perform an autopsy to collect various metastatic organs, including the brain, heart, lungs, liver, spleen, kidneys, lymph nodes, female reproductive system (female mice), bladder, and bones. See an example in Figure 4.
  c. Spray the mouse with 70% Ethanol.
  d. Make an incision from the abdominal site and cut into subcutaneous layer.
  e. Expand the incision to expose the inner organs, see an example in Figure 4B (left panel).
  f. Remove the intestine, see an example in Figure 4B (middle panel).
  g. Separate the organs in the order of liver, spleen, kidneys from the abdomen.
  h. Lift the xiphoid with the tweezer, cut the chest open.
  i. Lift the trachea, and harvest the lungs and heart.
  j. Search and harvest enlarged lymph nodes including axillary lymph nodes, abdominal lymph nodes, and paraspinal lymph nodes.
  k. Decapitate mouse and collect the brain.
  l. Store all the organ in the ice-cold PBS during collection.
22. Fluorescence imaging (Figure 5).
  a. Fix metastatic organs immediately after collection in 10% neutral buffered formalin for 24 h at 4°C for 16 h.
  b. Replace the formalin with 70% Ethanol.
  c. Image the organs by fluorescence microscope.
    i. Select the right fluorescence channel and bright-field channel.
    ii. Use the lowest magnification to obtain the whole picture of the organs.
    iii. Remove the organ from the 70% ethanol solution and gently tap the organ on tissue to remove excess ethanol solution.
    iv. Gently place the organ in a 100 mm Petri dish with a tweezer.
      **CRITICAL:** Do not squeeze the organ. Pick up the organ gently with the tweezer.
    v. Take a picture with bright-field and fluorescence channels.
      ***Note:*** Use the bright-field channel to focus on the organ surface and use the fluorescence channel to search for positive metastatic nodules.
    vi. Flip to the other side of the organs for liver, lungs, and kidneys.
    vii. Image the organs.

**Figure 5:**
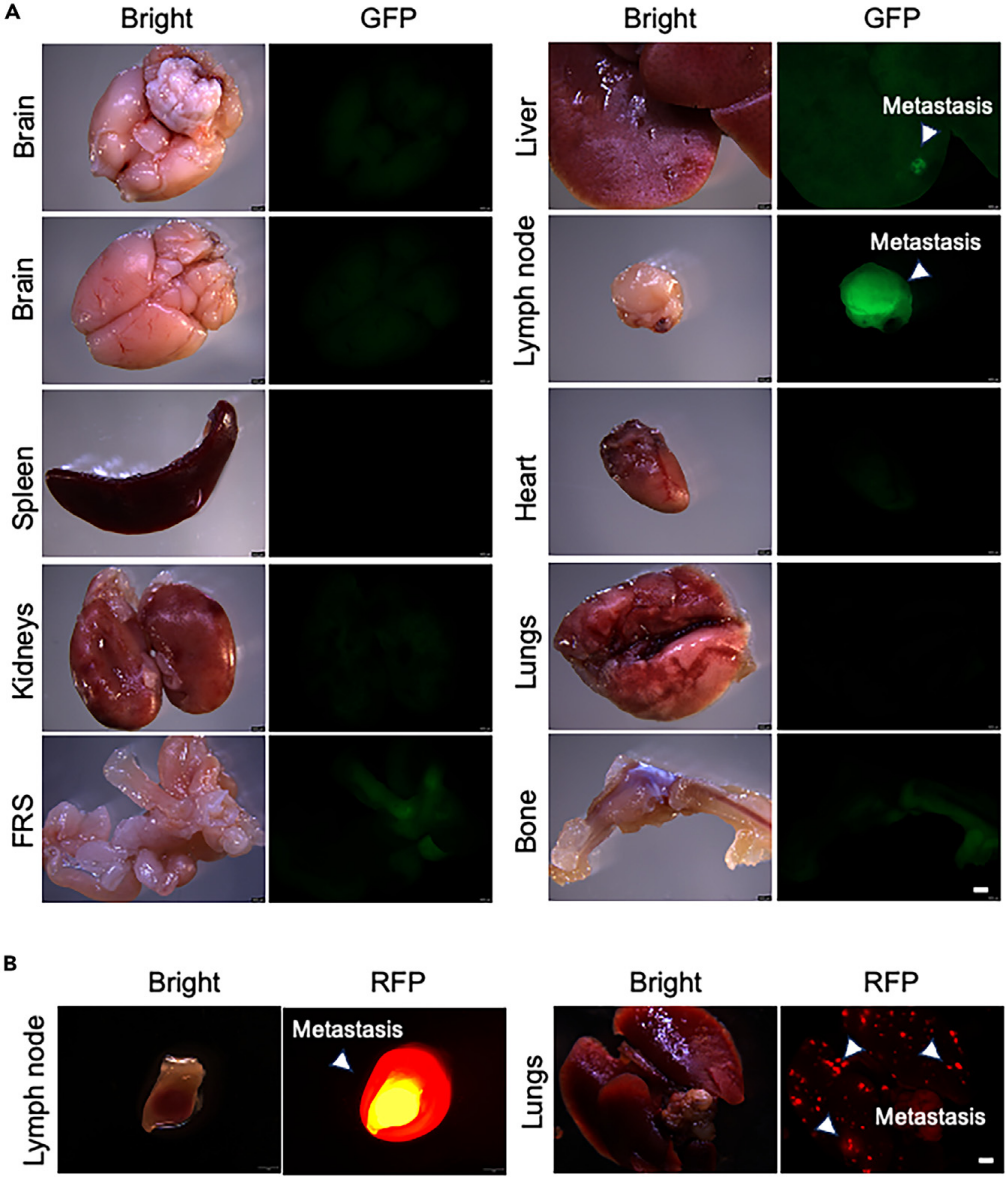
Fluorescence imaging of diverse organs from mice with spontaneous metastasis (A) Representative fluorescence imaging of organs harvested from mice with NCI-H82-GFP-Luc metastasis model. Scale bars = 2 mm. (B) Representative RFP imaging of a lymph node and lungs harvested from mice with DU-145-RFP-Luc metastasis model. Scale bars = 2 mm. White arrows indicate metastatic nodules.
23. Histological and molecular biological analysis (Figure 6).
  a. Process the organ by tissue processor.
  b. Embed tissues in paraffin blocks.
  c. Section into 4–5 μm thick sections.
  d. Stain tissue sections with hematoxylin and eosin (H.E.) for histological analysis.
  e. Perform immunohistochemistry (IHC) or immunofluorescence (IF) staining to evaluate the expression of specific markers.

**Figure 6:**
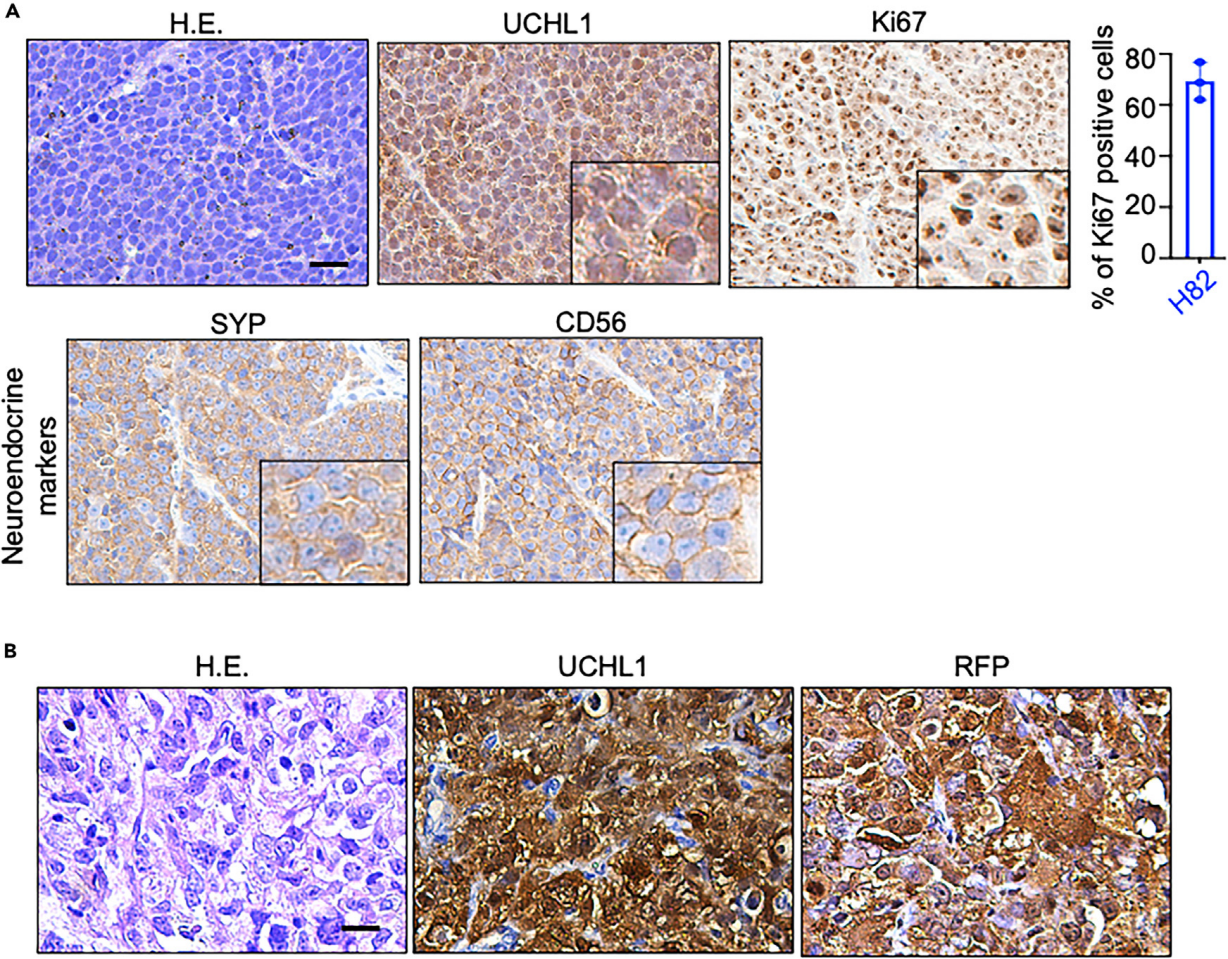
Histological analysis of harvested primary tumors (A) H.E. staining and IHC staining of UCHL1, Ki67, and neuroendocrine markers including SYP and CD56 in NCI-H82 primary tumors. The percentage of Ki67-positive cells was quantified. Scale bar = 20 microns. (B) H.E. staining and IHC staining of UCHL1 and RFP in DU145 primary tumors. Scale bar = 20 microns.

***Note:*** Perform IHC for a proliferation marker (Ki67), markers associated with neuroendocrine differentiation (synaptophysin, CD56, and chromogranin A), and the target of interest (UCHL1) (Figure 6). Stain for RFP or GFP to further confirm the presence of tumor cells (Figure 6).

24. Quantification of metastatic burden.
  a. Count the number of metastatic lesions in each organ using fluorescence images by ImageJ.
    i. Open the photo with ImageJ.
    ii. Change the image type to 8-bit.
    iii. Adjust the threshold and click “Apply”.
    iv. Select “Analyze with particles” in the “Analyze” tab.
    v. Record the number of the metastatic nodules.
  b. Quantify the area of metastatic nodules in organs using ImageJ.
  c. Calculate the metastatic burden as the percentage of the total organ area occupied by metastatic lesions.

## Expected outcomes

Using this protocol, we generated NCI-H82-GFP-luciferase and DU145-RFP-luciferase spontaneous metastasis models using 1 × 10^6^ cells to form primary tumors (Figure 1). The primary tumors were palpable 2 weeks after subcutaneous injection (Figure 2). After measuring NCI-H82 xenograft for 2 weeks and DU145 xenograft for 6 weeks, the average primary tumor volume reached 400 mm^3^ (Figure 2). Both models developed spontaneous metastasis with detectable bioluminescent signals at 3 weeks post-surgical removal of the primary tumors (Figure 4). Based on our experiences, we detected 100% metastatic incidents based on red fluorescence signals on diverse organs by fluorescence microscope imaging in the DU145-RFP-luciferase model and 86% metastatic incidents in the NCI-H82-GFP-luciferase model.

In our protocol, the main metastatic organ was lymph nodes in spontaneous metastasis models of both cancer types (Figure 5). Liver, lungs, and kidneys were also metastatic organs observed in the subcutaneous spontaneous metastasis models (Figure 5). The experimental endpoint varies based on different cell lines, cancer types, and mouse status. At the endpoint, diverse organs were harvested for further imaging and histological analysis. By combining imaging techniques and histological analysis, we further quantified the metastasis incidence and metastatic burden. Fluorescence imaging revealed the positive metastasis signals in lymph nodes, liver, and lungs (Figure 5). In addition, H.E. staining showed the morphology of tumor xenografts, and immunochemical staining demonstrated high expressions of UCHL1, Ki67, and NE markers including SYP and CD56 (Figure 6). IHC staining of RFP demonstrated the presence of DU145-RFP tumor cells in tumor tissue (Figure 6).

The protocol presented a comprehensive framework for studying spontaneous metastasis in mice. Testing gene function and therapies in cancer can be incorporated into this protocol. Future studies utilizing this model may lead to the identification of therapeutic targets and the development of therapeutic strategies for patients with metastatic cancer.

## Quantification and statistical analysis

The number of metastases can be quantified by counting the RFP or GFP foci. Quantification of the number and size of metastasis nodules was performed by ImageJ. See an example of the quantification, refer to Liu et al.^1^

## Limitations

One major limitation of this spontaneous metastasis model is that the mice may not develop metastasis on a 100%. The success rate of developing spontaneous metastasis and the patterns of metastatic spread vary based on cancer cell lines and cancer type. Pilot studies using different cell lines and testing different endpoints can help with future experimental design. Although spontaneous metastasis models recapitulate the nature metastatic process, there are limitations associated with long experimental duration between the formation of primary tumors to the detection of positive metastases. The time required for metastasis to occur and become detectable in major metastasis organs might not align with the progression observed in patients. Based on our experience, the spontaneous metastasis model had a much lower metastatic burden compared to the intracardiac injection metastatic colonization model and the tail vein injection metastatic colonization model. Nevertheless, the spontaneous model is a widely used tool in cancer research, providing valuable insights into the biology of metastasis.

## Troubleshooting

### Problem 1

Low primary tumor incidence (related to steps 1–11).

### Potential solution

• Ensure high viability of the cancer cell lines before the implantation.
• Make sure to use immunocompromised mice for human cells.
• Implant a higher number of cells to establish the primary tumor.
• Ensure the injection technique is correct, which is essential for accurate delivery of tumor cells.

### Problem 2

Unhealthy mice 24–72 h post-surgery (related to steps 17–18).

### Potential solution

• Reduce surgical time and anesthesia time.
• Make sure there is no internal bleeding or organ damage during surgery.
• Ensure all procedures are performed under strict sterile conditions to avoid infection. Apply antibiotics to the surgical area if needed.
• Separate mice post-surgery to reduce stress and the possibility of fighting.
• Provide post-surgical care including wound care, pain management, and sufficient monitoring.
• Consult with veterinary staff for the most supportive care.

### Problem 3

Observing recurrent tumor on the primary tumor site (related to steps 19–20).

### Potential solution

• Ensure the primary tumor is fully removed with the tumor membrane.
• Make sure to cut off any skin attached to the primary tumor.
• Cauterize all the blood vessels before resecting the tumor.
• Perform bioluminescence imaging post-surgery to confirm complete tumor resection.
• Do not calculate the bioluminescent signal from the recurrent tumor.

### Problem 4

None or Low bioluminescent signal at 4 weeks post-surgery (related to 19–20).

### Potential solution

• Detecting metastatic lesions can be challenging if the imaging technology is not sensitive enough, leading to underestimations of the metastatic burden. Longer bioluminescence imaging time may be needed. Adjust the position of the mice to obtain stronger signals.
• Increase the cell number for primary tumor injection.
• Remove the primary tumor at larger average tumor size in future experiments.
• Choose more aggressive cancer cell lines with higher invasive abilities.
• Run a pilot study to test the best experimental endpoint.
• The immune system of the host may limit the spread of cancer cells. Make sure to use immunocompromised mice for human cells.

## Resource availability

### Lead contact

Further information and requests for resources should be directed to and will be fulfilled by the lead contact, Tanya Stoyanova (tstoyanova@mednet.ucla.edu).

### Technical contact

Technical questions on executing this protocol should be directed to and will be answered by the technical contact, Shiqin Liu (ShiqinLiu@mednet.ucla.edu).

### Materials availability

All luciferase and fluorescence-positive cancer cell lines generated in this study are available from the lead contact with a completed materials transfer agreement. This study did not generate new unique reagents.

### Data and code availability

This study did not generate or analyze datasets. This study did not generate custom computer code.
